# Designing a 'NHS friendly' complementary therapy service: A qualitative case study

**DOI:** 10.1186/1472-6963-8-173

**Published:** 2008-08-12

**Authors:** Lesley Wye, Alison Shaw, Debbie Sharp

**Affiliations:** 1 Academic Unit of Primary Health Care, University of Bristol, 25 Belgrave Road, Bristol BS8 2AA, UK

## Abstract

**Background:**

Provision of complementary therapy services within the NHS is scarce and contested. However, their adoption may be more likely in a service model that is designed to the specifications of clinicians and Primary Care Trust (PCT) managers. Our objective was to identify the features of a 'NHS friendly' service to inform service designers who wish to develop NHS complementary therapy services.

**Methods:**

Using a case study approach, two sites offering complementary therapies on NHS premises were studied using interview and documentary data. We conducted interviews with 20 NHS professionals, including PCT managers and clinicians. We used descriptive content analysis to analyse interview data. We collected and analysed documentation, such as referral data, funding bids and evaluations, to compare reported and documented behaviour.

**Results:**

Ideally, a 'NHS friendly' complementary therapy service should offer a limited number of therapies for a specific condition for high priority patient populations (e.g. acupuncture for addictions). In this service model, the therapies should be perceived to have 'good' evidence for conditions where there are 'effectiveness gaps' (i.e. current treatments are limited). The service should be evaluated and regularly promoted. Inter-professional relationships would flourish through opportunities for informal contact and formal interactions, such as observations of consultations. However, the service should include gatekeeper mechanisms to control demand and avoid picking up 'unmet need' (i.e. individuals currently not accessing NHS services). The complementary therapy service should pay for itself and reduce NHS costs elsewhere, such as hospital admissions.

**Conclusion:**

The service design model identified in this study is problematic. For example, it is contradictory to provide specific interventions for specific conditions within a holistic healthcare framework. It is difficult to avoid providing for 'unmet need' while concurrently filling 'effectiveness gaps'. In addition, demonstrating the impact of a community service on reducing hospital admissions is challenging. Those seeking to establish a NHS complementary therapy service might be well-advised to meet as many of the criteria of a 'NHS friendly' model as possible, recognising that its full realisation may be impossible. However, during periods of innovation and financial security, some relaxation of expectations may occur.

## Background

Current provision of complementary therapy services in the NHS is patchy, sporadic and under threat [1,2]. However, their adoption may be more likely with a service model that is designed to the specifications of clinicians and Primary Care Trust (PCT) managers. Although many models of complementary therapy services exist, the details of service design have rarely been comprehensively studied, with one notable exception [3]. Most of the existing literature tends to focus on evaluations of particular services [4-6] or ways in which doctors, nurses and complementary practitioners might work together [7-13]. The aim of this study was to specify the features of a 'NHS friendly' complementary therapy service – that is a service that would be accepted, endorsed and supported by NHS stakeholders such as clinicians and PCT managers – to help those who are considering ways to design such services in NHS primary care.

Research on healthcare professionals' attitudes and referral behaviours regarding complementary therapies should offer some guidance on the design features of state funded complementary therapy services. These studies are abundant, particularly for doctors and nurses [14-23]. But they take self-reported behaviour at face value, assuming that people do as they say, so unsurprisingly, different studies have given different results [24]. Moreover, relying on studies of doctors and nurses alone neglects the views of commissioners, such as PCT managers, who allocate funding for NHS healthcare. With recent national policy initiatives, this group is becoming increasingly powerful within the NHS [25]. The few studies of commissioners that do exist have reported that evidence of effectiveness of complementary therapies is strongly persuasive [26-28]. But this might only partially explain the absence of complementary therapy services in the NHS, as complementary therapy treatments such as spinal manipulation (osteopathy and chiropracty) for mechanical neck disorders [29], acupuncture for headaches [30,31] and herbal remedies for benign prostate hypoplasia [32,33] are not widely available in the NHS, despite having relatively robust evidence of clinical, and in some cases, cost effectiveness.

As there appeared to be little practical guidance available on service design for those interested in developing NHS complementary therapy services, we undertook a study to address this. We have identified the features of an 'ideal' NHS complementary therapy service in primary care. But this comes with two caveats. First, NHS local health systems are highly context specific and therefore a 'one size fits all' blueprint is not realistic. Thus, the design features detailed in this study are suggestions only. Second, this paper does not address the question of 'should complementary therapy services be mainstreamed within the NHS?' Instead, our question was – what are the features of a NHS service offering complementary therapies that would be more acceptable to primary care doctors, nurses and PCT managers? Or, in other words, what does a 'NHS friendly' complementary therapy service look like?

## Methods

### Study design and case site selection

This study was undertaken by three health services researchers, two with clinical backgrounds (DS is a GP; LW is a complementary therapist) and one with a social science background (AS). We chose a case study approach, as data can be collected from and compared across multiple sources. Because previous studies had over-relied on interviews, we wanted to include other forms of data such as documentation to check reported behaviour against documented behaviour. This research was part of a larger study exploring the changes necessary for the incorporation of complementary therapies into NHS primary care.

We chose two sites where complementary therapy services were provided on NHS premises. We focused on NHS premises, as we believed clinicians and PCT managers who had direct experience of complementary therapy services had greater knowledge of such services and would be better placed to comment knowledgeably on service design features. Site selection was based on:

• Funding source (e.g. NHS or other government funding).

• The degree to which the service appeared to be valued by NHS professionals (as determined through initial contact in recruitment).

• Willingness to take part (two other sites declined).

The first case site included a complementary therapy service that was funded by New Deals for Communities money from the Office of the Deputy Prime Minister. This was part of a national urban regeneration programme, in which local communities bid for funds to set up a range of projects in health, education, crime and safety and housing. In this particular community, local residents opted to spend some of the funding on creating a low cost, local complementary therapy service. Fifteen therapists offered a range of different therapies including reflexology, osteopathy and acupuncture. Two local GP surgeries provided treatment rooms at no cost. Patients could be referred to the service through any local health professional (GP, practice nurse, health visitor, midwife, addictions counsellor) and self-referrals were also accepted. As the service was subsidised by New Deals for Communities, patient fees were minimal, initially £5 for the employed and £3 for the unemployed. After nearly four years of funding, New Deals for Communities money finished as it was time limited. In 2006, the local PCT (NHS) took over the funding of a radically modified complementary therapy service that offered three therapies (osteopathy, chiropracty and physiotherapy) for musculoskeletal conditions only. Instead of eight sessions, patients were now only entitled to two and self-referrals were no longer accepted.

Having completed fieldwork at the first case site and concluded that the service was not popular with some of the doctors, nurses or PCT managers that we had interviewed, we purposefully selected a second case site with a complementary therapy service that appeared better utilised and more highly regarded by referring clinicians. This complementary therapy service was part of a city-wide women's health service that provided treatments for women with pre-menstrual syndrome and menopause. As an adjunct, the women's health service also provided homeopathy, reflexology and aromatherapy treatments which were delivered by two medically trained professionals (a doctor and a nurse) and one professional therapist. To receive complementary therapy treatments, any patients with menopausal or pre-menstrual syndrome symptoms could self-refer into the women's health service or be referred by any NHS clinician across the city (e.g. GP, practice nurse, district nurse, health visitor). After initial assessment by one of the three specialist doctors in the women's health service, these doctors then referred on patients who could not have or did not want pharmacological treatments to the complementary therapy service. Only these three specialist doctors could refer patients to complementary therapists within the complementary therapy service.

The complementary therapy service at this case site had been funded and line managed by the NHS since 1998. It was located in a former community trust in an inner city area in England. All therapists had NHS contracts. Shortly after beginning fieldwork for this study, the reflexology/aromatherapy service was discontinued as the therapist retired and so fieldwork focused primarily on the homeopathy service. More unexpectedly, NHS funding for the homeopathy service ceased in the summer of 2006, just months after completing fieldwork, as a result of local and national financial cutbacks on 'non-essential' services.

### Data collection and analysis

We used purposeful sampling techniques to select a range of participants for interviews at each case site [34]. A key criterion was professional background (doctor, nurse, administrator or PCT manager). Although their views are valuable, patients and therapists were not included in this particular study, as we were not focusing on the features that would make such a service acceptable to therapists and patients, but instead wanted to find out what features would be acceptable to NHS doctors, nurses and PCT managers. Further criteria guiding sampling were frequency of referral to the complementary therapy service (high or low for clinicians only), current or past key role in developing, maintaining or delivering the service (administrators, PCT managers, clinicians) or senior managerial positions such as Chief Executive (PCT managers). One administrator (practice manager) refused to be interviewed at the first case site, but all those approached at the second case site agreed. Sampling selection at the first case site stopped when no new themes were arising and at the second when all eligible study participants had taken part.

In total, we interviewed 20 NHS professionals across the two case sites. These included: five PCT managers, nine doctors (six GPs and three women's health specialist doctors), four nurses (two practice nurses, one health visitor and one women's health specialist nurse) and two administrators (one practice manager and the administrator for the women's health service). All of the PCT managers interviewed were based in the local PCT and included a Chief Executive, a Chair, two Public Health specialists and a pharmacist. The doctors, nurses and administrators were based at the GP surgeries (case site one) or community trust (site two). The NHS professionals we interviewed were either influential in decisions about funding the complementary therapy service (PCT managers), provided administrative support to the service (administrators) or were eligible to refer into the service (doctors and nurses).

We devised a semi-structured topic guide, which we reviewed before every interview. Topics included: knowledge and personal use of complementary therapies, attitudes towards complementary therapies and influences on those attitudes, experiences and opinions of NHS complementary therapy services, perceived referral behaviour (clinicians only), funding considerations (PCT managers only) and possible improvements to the service. Interviews lasted between 15 and 75 minutes and were audio-taped and transcribed verbatim.

We used descriptive content analysis to analyse interview data [35,36]. Aided by Atlas-ti software, we coded the interview transcripts using both anticipated codes from previous literature and more emergent codes that arose from the data. We coded transcripts in batches following completion of a series of interviews (e.g. PCT managers at site one, doctors at site two etc.). The coding framework developed as the interviews and analysis progressed, with codes being modified or merged, as appropriate, to account for new data. We re-coded all transcripts with the final coding framework, once fieldwork ended, to ensure more recently developed codes were universally applied. In addition to coding within Atlas-ti, we summarised each transcript into a single document noting significant points and quotations to better understand the overall 'story' of each interview.

From these two processes (that of Atlas-ti coding and summarising each transcript), we developed key themes. To elaborate our understanding of these themes, and check reported behaviour against observed behaviour, we also collected and analysed documentary data.

Documentary data included: service evaluations, funding bids, minutes of meetings (e.g. of the steering group overseeing the development of the service at site one), e-mails from PCT managers and referral databases of the services. We read service evaluations, funding bids, meeting minutes and e-mails. Information pertinent to service design were highlighted (e.g. referral pathways, service improvements, service design ideas, funding conditions and decisions). With these data, we identified both consistencies with interview data (e.g. indications for referral) and inconsistencies (e.g. the role of research evidence in funding decisions), which will be elaborated in the results section.

In terms of referral data, the first service held an Excel database that generated data on referrals resulting in treatment from 2001 to November 2006. Details included name of referrer, role (e.g. doctor, nurse etc.), therapy referred to and number of patients referred. At the second service, data were recorded manually from March 2004 – June 2006 and we collected details on name of referrer, therapy referred to and date. To analyse both these datasets, we calculated the frequency of referrals and type of therapy referred to per referrer. We used these data to check the reported behaviour of clinicians against their documented behaviour. For example, during interviews the two practice nurses stated that they were in favour of NHS funded complementary therapy services and were enthusiastic about the complementary therapy service located in their surgeries. Yet on checking against the referral database, neither had referred a single patient to the service. This then fed into interpretation of their interview data in teasing out what service features encourage clinicians to refer.

We further interrogated emergent findings from interview and documentary data by searching for negative cases, drawing mind maps and 'brainstorming' (see Riley 2000 for an explanation of brainstorming qualitative data [37]). To enhance reflexivity, the lead researcher made journal entries weekly or fortnightly. We tested 'face validity' [38] in seminars and conferences as well as individual meetings with a therapist, a PCT manager, a GP and professionals from the Foundation for Integrated Health.

The study received ethical approval from the London Multi-centre Research Ethics Committee in 2004. We began fieldwork in July 2004 and continued until June 2006. We assured all study participants that their responses would be anonymous and confidential and all gave written consent to participating in the study.

## Results

**Figure 1:**
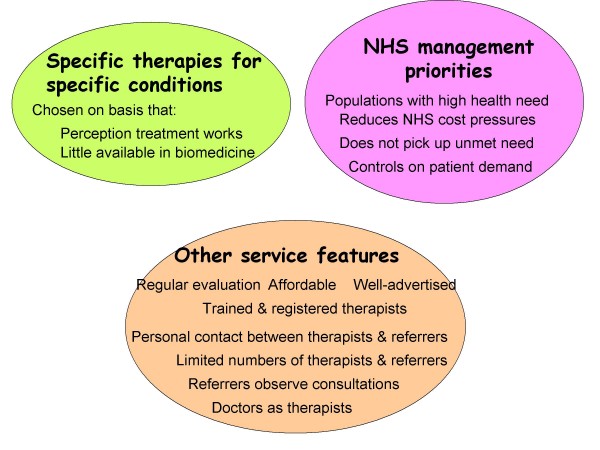
Service model characteristics of a 'NHS friendly' complementary therapy service.

In identifying the characteristics of a 'NHS friendly' complementary therapy service, the service design features identified have been grouped into three areas, as detailed in the following diagram. Each will be discussed in turn in the results section (see Figure 1).

### Specific therapies for specific conditions

During interviews, PCT managers and NHS clinicians appeared to be more favourably disposed towards a service model whereby specific treatments were provided for specific conditions (e.g. acupuncture for pain or chiropracty for low back pain). This 'specific condition' service model existed at the second case site, which was funded by the NHS, as three therapies (aromatherapy, reflexology and homeopathy) were provided for two types of female hormonal conditions. Once the NHS took over funding the complementary therapy service at the first case site, this type of model also was put in place with three therapies offered for musculo-skeletal conditions only. Thus, a service model in which any complaint would be treated (such as the service at case site one when it was funded by New Deal for Communities) appeared less popular. As a senior PCT manager said,

I think any introduction of complementary therapies has to be incredibly disciplined. And so we need very clear protocols that say this is the sort of case, this is the sort of need that we're going to meet through this service. Not a' come all ye'. (PCT manager BC site 1)

Another PCT manager gave an example of how a targeted complementary therapy service could work in practice.

Acupuncture, we know that in other agencies it's used in helping people over drug misuse and alcohol misuse. It's known to be, or it's been shown to be, quite effective. So if it was being targeted at those people, then we'd say well that is something we would want to support.... But we're not likely to develop an enhanced service for complementary therapy. It would be an enhanced service for a specific condition which may include complementary therapy as part of the service. (PCT manager CC site 1)

However, selection of the 'right' therapies and specific conditions is crucial. Our data suggest that one criterion for the selection of therapies and conditions in a 'NHS friendly' complementary therapy service is that NHS professionals believe little or nothing else is available.

I mean as a medic, it's sometimes quite hard to understand it [homeopathy] ....But I'm prepared to go with it and just, because I know I can't do much more from the normal medicine point of view. (Doctor Y site 2)

This has been coined as an 'effectiveness gap' [39,40]. But such a gap is not sufficient in and of itself. According to the study participants interviewed, the 'gap' condition chosen should impact on a substantial numbers of patients. For example, a PCT manager partly attributed the success of the mainstreaming of counselling to the perception that counselling is appropriate for large patient volumes.

PCT managers and NHS clinicians in this study stated that another criterion for therapy and condition selection is 'good' evidence of clinical effectiveness. But interestingly, this appeared to be based more on perceptions of research evidence than actual knowledge. For example, only three of the nineteen NHS professionals interviewed had directly accessed the research literature on complementary therapies. Their opinions on which complementary therapies had good evidence appeared to be based on collective, unchallenged perceptions, rather than grounded in fact based knowledge.

For example, data from steering meeting minutes and interviews at case site one indicated that therapies should be chosen on the basis of robust evidence. Herbal medicine was rejected, because clinicians believed that there was not any research evidence, while other therapies such as reflexology and aromatherapy were adopted. Yet, the evidence base for herbal medicine has been identified as the strongest amongst complementary therapies [41], while the research evidence for reflexology and aromatherapy is less robust. Moreover, a doctor at the first site, who claimed during interview that her decisions were based on evidence and she believed that there was insufficient evidence for complementary therapies, was the third highest referrer of the 24 doctors to the complementary therapy service, according to the referral database.

We also found discrepancies in PCT managers' reported positions that research evidence was paramount in the decision process. For example, although PCT managers claimed that research evidence was an essential precursor to NHS funding of the complementary therapy service, the successful PCT funding bid for the revamped service in 2006 included no reference to evidence of therapeutic or cost effectiveness. An e-mail from the relevant commissioning manager confirmed that such evidence was not needed. Similarly, the funding bid for the service at the second case site in 1998 cited an audit study on homeopathy for one type of female hormonal condition [42] and a more general systematic review on homeopathy [43]. No specific randomised controlled trials on homeopathy for menopause or pre-mentrual syndrome or any research on aromatherapy or reflexology were referenced as research evidence in this bid. Thus, factors other than research literature appeared to be influencing both perceptions of 'good' evidence and referral and funding decisions for clinicians and PCT managers.

### NHS management priorities

In addition to developing a 'specific condition' model, a 'NHS friendly' complementary therapy service design should address NHS management priorities. One current NHS management priority is targeting high health need populations. A doctor at the second case site acknowledged during interview that the service had targeted a low NHS priority group (women with pre-menstrual syndrome and menopause symptoms).

Everyone in the NHS, they're looking much more at teenage pregnancies and all those figures that are figures, whereas the menopausal lady doesn't come high in the profile in the NHS.... She's not a target, she's not a number, she's not anything. She's very important to the family and everything else at home, but not in the NHS, so in the scheme of things we're very much out on a limb really. We're low priority is the [women's health] clinic, and so homeopathy is probably even lower in that it's just an extra to our clinic. (Doctor Y site 2)

This may have contributed to the decommissioning of the complementary therapy service at the second site, although we cannot be sure as these events took place after fieldwork terminated.

Another crucial NHS management priority identified in interviews is reducing costs.

Well, the PCT is in a very difficult financial position at the moment. We have what's called a local delivery planning group, which is the sort of first sound bite where bids for funding would go. And the criteria we use will be – how much money is this going to save? Basically, it has to pay for itself in terms of hospital admissions or even make savings over and above the cost of running the service. (PCT manager CC site 1)

So ideally, a 'NHS friendly' complementary therapy service should either pay for itself (cost neutral) or demonstrate that less money can be spent elsewhere in the NHS (ideally on hospital admissions), as a result of the complementary therapy service (cost saving). By demonstrating a cost saving such as reduced medication, GP consultation or hospital usage, complementary therapy services can help shift the allocation of financial resources into community services.

We can only invest if we find things we can disinvest in. Now that mainly is disinvesting in hospital interventions, whether it be outpatient clinics or diagnostics...And if we can reduce those because we're doing an earlier intervention in primary care, then we can for the first time probably take money out of the acute hospital system and bring it into the community. (PCT manager CB site 1)

But, according to PCT managers, to save costs, NHS complementary therapy services should only target patients who are already receiving NHS treatments rather than those currently outside the system.

If we had the respiratory nurse we'll probably find more people with wheezes and so being seen that wouldn't otherwise have been done because they wouldn't have been serious enough to get a hospital appointment.... Now that's an issue for us about whether we're expanding the boundaries of NHS capability and NHS priorities because we're making it more available. That's already an issue for us and I think there is a concern that complementary therapies would take that even further. (PCT manager BC site 1)

So, for example, a patient with low back pain having physiotherapy is a legitimate focus for a NHS complementary therapy service, as hospital costs for the out-patient physiotherapy service are incurred and a NHS complementary therapy service may be able to treat the same complaint as effectively and more cheaply. But an individual with the same condition who is 'self-managing' is not a candidate for a 'NHS friendly' complementary therapy service, as he or she does not currently make demands on hospital services or GP practices and thus incurs no costs. Ideally, complementary therapy services should treat the former (i.e. those requesting hospital and GP practice services), but avoid the latter (i.e. those who are self-managing).

Known as 'picking up unmet need', the widening of the NHS net is a major concern to PCT managers given its financial and resource constraints. Thus, PCT managers need reassurance that a 'NHS friendly' complementary therapy service will either be cost neutral or cost saving and reduce demand on existing services, rather than create a new pool of patients that incur additional costs. PCT managers received this reassurance with the service at case site two, as patients were either under the care of the specialist women doctors or received complementary therapy treatments. However, patients at the first site could access both conventional and complementary therapy services in tandem.

An additional NHS management concern is that demand for complementary therapy services will outstrip supply. Consequently, a 'NHS friendly' complementary therapy service will have mechanisms to regulate demand. These mechanisms were incorporated at the services in both case sites. Funding bids showed that patients could have up to eight treatments at the complementary therapy service at the first case site and patients could have up to six at the service at the second case site. Consultation length was also limited. Therapists at the service at the first case site offered appointments of 30–60 minutes for all consultations, while the homeopaths at the service at the second case site spent an hour with new clients and 15–20 minutes in follow ups. At the service at the second case site, an additional control mechanism was the utilisation of doctors as gatekeepers to the complementary therapy service.

I think it works, I think it's essential. It's a screening process in that it does control who gets to the homeopathist [sic] and there's some feedback, so we've got a history and some idea of what's going on. (Doctor Y site 2)

### Other service features

As well being a 'specific condition' service model and meeting NHS management priorities, a 'NHS friendly' complementary therapy service will have other notable characteristics.

For example at the services at both sites, evaluations had been conducted. Other research we have conducted found that the impact of complementary therapy service evaluations on funding decisions was limited [44]. Nonetheless, during interviews, study participants stated that they believed regular service evaluation was important.

I think [this] work has been appreciated only because we do the evaluation and [the therapist] continues to do the evaluation. And I think that's very, very powerful. (Doctor NP site 2)

In addition, doctors and nurses, in particular, stressed that a 'NHS friendly' complementary therapy service should be affordable. At the service at the first site, clinicians were "put off" referring (Doctor PS site 1) when patient contributions increased from £3 and £5 (unwaged and waged respectively) to £5 and £10–£15, as New Deals for Communities funding drew to a close.

Clinicians also stated in interviews that they were unlikely to refer if they did not know they could. With constant NHS staff turnover and a service based across two surgeries, ensuring a high profile for the service at the first site was challenging. Moreover, some potential referrers to the service at the first site indicated they needed personal contact as well as promotional literature to feel confident about referring patients.

Now it may be because of my part time role at [surgery], but I haven't met any of the [service] therapists and I like to meet people and then I think I'd feel much more comfortable about saying, "You know what I think? You should go and see [X] about this. Why don't we arrange a referral and this is how we can do it." (Doctor PS site 1)

This was more manageable at the second site partly because only six individuals were involved (three referring women's health specialist doctors and three therapists) unlike the service at the first site where there were 15 therapists and over 100 potential referrers. As one doctor from site one explained during interview,

You know we refer but part of the problem is that there are tons and tons of therapists many of which do few hours. It's hard for us to get to know any of them particularly well and build up a professional relationship. (Doctor DF site 1)

Meeting therapists might also go some way to address NHS professionals' concerns about safety. During interviews, concerns about safety were usually expressed in terms of 'safe therapists' rather than 'safe therapies'.

I would just like to know that hopefully they are not abusive relationships. And you can have an abusive relationship with a mainstream clinician, so I don't excuse general practitioners from that either. But I would hope that on the whole a lot of these people are very vulnerable and it worries me if they are going to see somebody who has had no training and no registration. (Doctor PS site 2)

To address the safety issue, data from minutes of meetings at site one and interview data from both sites indicated that only trained therapists registered with a recognised professional body would be employed at the services.

Concerns about safe therapists may also have been addressed at the second site by employing a doctor as a therapist. As one doctor at the second site stated during interview,

I'm a little bit more comfortable with [the medical homeopath] having a medical background as well as a homeopathy background. (Doctor Y site 1)

The induction process at the second site also helped to reduce fears around safety and cultivate inter-professional relationships, as all new doctors and nurses to the women's health service observed homeopathy consultations.

Whenever I first came along to do this [women's health] work, I actually spent time with [homeopath] and sat in with her, seeing her patients and I think it is really valuable that because it gives you an understanding of actually how the homeopath works, you know, how they really go through their decision making process. (Doctor WL Site 2)

Doctors and nurses at the service at the first site, where inter-professional relationships were less well developed, did not observe complementary therapy consultations. Nor, after nearly four years of complementary therapy service provision, did these NHS clinicians have much more understanding of complementary therapies.

I don't think I actually know any more about complementary therapies really. (Doctor BM Site 1)

So to sum up, a 'NHS friendly' complementary therapy should incorporate regular evaluation and be affordable and well-advertised. PCT managers and NHS clinicians need to know that the therapists working in a NHS complementary therapy service are 'safe'. This type of reassurance may come about through employing trained and insured therapists, as well as employing doctors as therapists, and through personal contact between therapists and potential referrers. Inter-professional relationships are more likely to flourish if the numbers of therapists offering treatments and the numbers of referrers are small and if there are opportunities for informal contact and formal interactions (e.g. observation of consultations).

## Discussion

In summary, a 'NHS friendly' complementary service would provide a limited number of therapies for specific conditions for patient populations of high priority where current treatments are limited, ineffective or non-existent. The therapies chosen would be perceived by NHS professionals as having 'good' evidence of effectiveness. In addition, services would be precisely targeted without picking up previously unmet need, cover their own costs and (ideally) demonstrate an appreciable impact on reducing NHS costs elsewhere. The service would be regularly evaluated, affordable and well-advertised. Furthermore, inter-professional relationships would flourish between 'safe' therapists and NHS clinicians, ideally through informal personal contact and more formal exchanges when doctors and nurses observed complementary therapy consultations.

### Strengths and limitations

This study has addressed a neglected issue by examining the ideal characteristics of a 'NHS friendly' complementary therapy service in primary care. However, a limitation of this study is that at the second site no PCT managers were interviewed, as service providers feared this would jeopardise the funding of the service by raising its profile. An additional limitation is that the study drew on only two case sites. But by focusing in-depth on two services, we were able to carry out detailed examination of reported and actual behaviour as well as expressed views. Further research, possibly through analysis of the Westminster mapping study [1], would clarify the extent of this 'NHS friendly' complementary therapy service model nationally.

### Implications

However, there are numerous difficulties with the 'NHS friendly' complementary therapy service model.

First, PCT managers and NHS clinicians insisted, when questioned, that 'good' evidence should determine funding allocation, therapy selection and referral practices, yet their behaviour contradicted this. This suggests that, in practice, factors other than actual evidence may have influenced the decisions of our study participants, as has been found in other studies [45-47]. Nonetheless, because the calls for evidence are so strident, the perception of 'good' evidence appears important. Thus, those designing a complementary therapy service have two choices: they can either work to foster the perception of 'good' evidence or select complementary therapies for which NHS professionals already believe 'good' evidence exists, such as acupuncture, osteopathy and chiropracty. Once clinicians and PCT managers are committed to developing a complementary therapy service, relatively little solid evidence may be required. For example, in our study an audit and a general systematic review were sufficient at the second case site to support the bid for homeopathy services. Elsewhere, in bidding for NHS funding in 2006, the Glastonbury Health Centre cited the UKBEAM trial [48,49] and this was enough to convince PCT managers to continue funding osteopathy and acupuncture for musculo-skeletal conditions [50].

A second difficulty is that a 'NHS friendly' model seriously challenges the holistic tenet common to so many complementary therapies. Working 'holistically' means attempting to balance individuals in their inter-related emotional, mental, physical and spiritual dimensions rather than focusing on alleviating a narrow set of predominantly physical (and occasionally mental) symptoms. In doing so, therapists see themselves as generalists. But in the 'specific treatments for specific conditions' service model illustrated in this study, therapists would be specialists, who use selected techniques and remedies to target particular complaints. So how can therapies be both holistic and selective? In Gibson's study of alternative practitioners working within the NHS, an osteopath recounted her difficulties in managing this conflict.

*The forms were divided up into neck problems, thoracic problems, lower back problems. We can't work like that, it's not osteopathic. For example, I'd get a patient with frozen shoulder [in addition to the referral problem] so start working on the shoulder – well you can't do that, that's a separate referral, that's breaking the rules *[51].

This troubled the osteopath sufficiently that she left NHS employment. Targeting treatments for specific conditions essentially conflicts with holistic healthcare philosophies.

A third difficulty with the service model is that of 'unmet need' and promoting self-care. Broadly speaking, study participants defined 'unmet need' as:

• Those suffering potentially dangerous conditions without knowing it

• Those aware of their condition but not seeking treatment either because they were managing it themselves or because they were ignoring their condition

• Those aware of their condition and seeking treatment but no services were available

• Those aware of their condition and seeking treatment, but available services were substandard or ineffective

For many therapists, those people who are self-managing (definition 2) are just the type of individuals for whom complementary therapies, with their emphasis on prevention and self-care, offer the greatest benefit. Instead of playing to the strengths of complementary therapies and offering these patients the extra resources they may require, a 'NHS friendly' service model discourages important self-management behaviours, which ironically are those currently promoted in national policies [52,53].

A fourth difficulty in a 'NHS friendly' service model is that the conditions treated should fill an effectiveness gap, defined as an area where current interventions are inadequate or non-existent (definitions 3 and 4). But the stipulation is that only those patients already within the system should be treated, and treated more effectively. If current NHS services are inadequate, patients may already be within the system, but if services are non-existent, they are likely to be outside. Likewise, those who are self-managing or unaware of their condition (definitions 1 and 2) are also outside the health system, but may be included once appropriate services are in place. PCT managers (such as BC in this study) are concerned that the funding of complementary therapy services will extend the already over-stretched boundaries of the NHS, as have other community service initiatives. In brief, the boundaries of the NHS would encompass patients meeting all four definitions of unmet need.

So how can complementary therapy services plug 'effectiveness gaps' while simultaneously only treating those already within the system? Providing more effective treatment for conditions currently not well treated will, by definition, create demand. This is obvious to everyone, including the pharmaceutical industry. In the Cooksey report on research funding, which was commissioned partly to review incentives to keep pharmaceutical industries within the UK, researchers are urged to identify and close "unmet needs" with medicines that open up new markets [54]. This implies that addressing "unmet needs" may be acceptable, as long as they are met with a pharmaceutical agent.

A final difficulty is that the 'NHS friendly' service model requires that complementary therapy interventions reduce costs elsewhere in the NHS. How many biomedical treatments are likely to meet this stringent criterion? By setting, or more importantly enforcing, such challenging standards, some NHS professionals are clearly indicating their reluctance to incorporate complementary therapies.

## Conclusion

The inherent contradictions in a 'NHS friendly' complementary therapy service model, and between this service model and the philosophies and approaches of many complementary therapies, make it problematic. Those seeking to establish a NHS complementary therapy service might be well-advised to meet as many of the stipulations of a 'NHS friendly' model as possible, recognising that its full realisation is inherently impossible. However, during periods of innovation and financial security, some relaxation of standards may occur and inroads made.

## Competing interests

The authors declare that they have no competing interests.

## Authors' contributions

Funding for the study was obtained by AS and DS. The study was conceived by LW. The majority of the data was collected and analysed by LW, with assistance from AS and DS, especially at the interpretative stages. LW drafted the manuscript and AS and DS made substantive revisions. All engaged in the design process, read and approved the final manuscript and gave approval for the final version.

## Pre-publication history

The pre-publication history for this paper can be accessed here:


